# Determination of the Basic Geotechnical Parameters of Blast-Furnace Slag from the Kremnica Region

**DOI:** 10.3390/ma16175966

**Published:** 2023-08-31

**Authors:** Roman Bulko, Soňa Masarovičová, Filip Gago

**Affiliations:** Faculty of Civil Engineering, University of Žilina, Univerzitná 8215/1, 010 26 Žilina, Slovakia; roman.bulko@uniza.sk (R.B.); sona.masarovicova@uniza.sk (S.M.)

**Keywords:** blast-furnace slag, geotechnical parameters, mineralogical analysis, California bearing ratio (*CBR*), oedometric modulus, shear strength of slag

## Abstract

A decisive aspect of site evaluation for construction is the presence of anthropogenic materials occurring in the geological environment. The geotechnical properties of blast-furnace slag were investigated as a potential substitute for aggregates in the construction industry. The basic geotechnical parameters of the slag were determined, which are critical for evaluating its stability, environmental impact, and usability in geotechnical construction. The research focused on monitoring the physical and mechanical properties of the two samples, and also included mineralogical analysis. The obtained results demonstrated that the slag belongs to the category of poorly graded gravel, *G*2*/GP*, and gravel with an admixture of fine-grained soil, *G*3*/G-F*. In addition, other important parameters, such as the water disintegration of the slag aggregate, the minimum and maximum bulk densities, the California bearing ratio (*CBR*), the oedometric modulus (*E_oed_*), and shear tests (the angle of internal friction *φ* and cohesion *c*), were determined. The results from this paper provide important information for the proper management of blast-furnace slag so to minimize its environmental impact and achieve sustainability in the mining industry. At the same time, it enables a better understanding of the use of slag as a substitute for aggregates in geotechnical tasks. Despite its local importance in relation to the investigated case, the presented study has significant educational and scientific value for the construction sector, where it is necessary to evaluate anthropogenic activities and materials.

## 1. Introduction

The dynamic development of construction infrastructure leads to a greater demand for aggregates, which are used for the construction of roads and buildings. The decline in natural-stone resources has led to the search for alternatives and replacements in the form of anthropogenic materials [1,2]. Among these anthropogenic materials, we recommend solid industrial waste from primary production, such as blast-furnace slag [3]. It is known from many studies that slag in contact with water undergoes volume changes and behaves in an unstable way. There are known examples of violations of the condition due to the penetration of moisture into the slag, or examples of the use of unsuitable slag [4,5].

**Figure 1:**
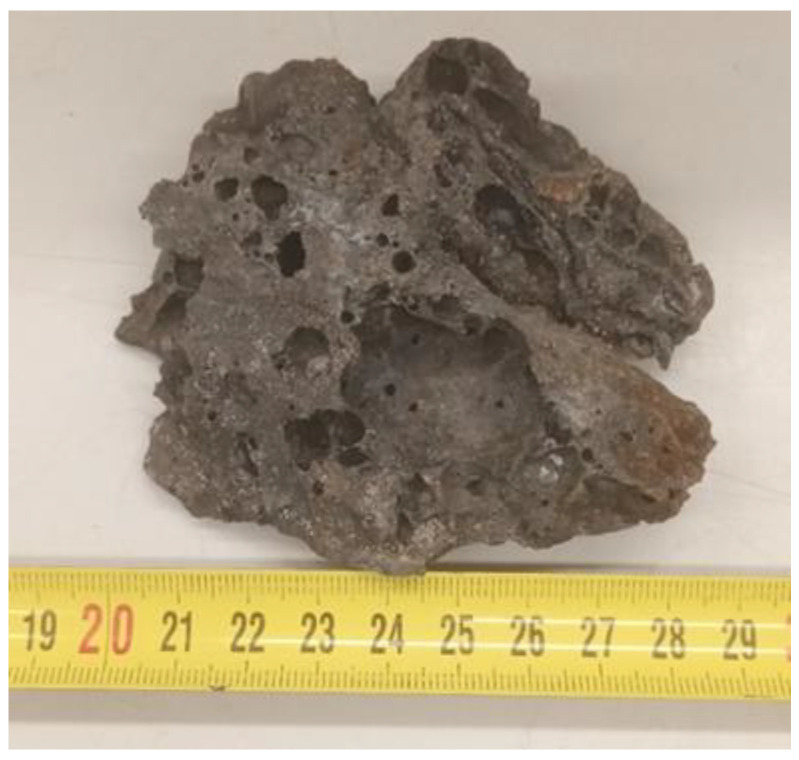
Porous structure of slag.

Slag differs from natural aggregates in its porous structure and cavities (Figure 1), and sometimes a sulfur smell can be detected. Blast-furnace slag is a dark gray to black, porous, and rough rock that contains various compounds, such as metal oxides, glass, and minerals. This material is very hard and durable, which makes it a suitable material for various construction and engineering purposes.

However, its composition, especially heavy metal minerals, are environmental pollutants [6,7,8,9,10,11]. Slag, as a secondary raw material, is widely used in the processing of concrete mixtures, and from the point of view of testing the applicability of slag in concrete constructions, research has been conducted by several authors [12,13,14,15,16,17]. Blast-furnace slag as an additive to concrete can improve its properties, such as its strength and weather-resistance, etc. Blast-furnace slag is also an economic alternative to natural building materials.

Blast-furnace slag mainly contains metal oxides, such as iron oxide (Fe_2_O_3_), silicon dioxide (SiO_2_), calcium oxide (CaO), aluminum oxide (Al_2_O_3_), magnesium oxide (MgO), titanium dioxide (TiO_2_), and manganese oxide (MnO) [18,19]. These oxides can be susceptible to volume changes when exposed to changes in humidity and temperature. It can lead to soil movement and the deformation of building structures if the slag is not handled with care and used properly.

At high temperatures, FeO can react with other components in the environment, and in the presence of oxygen, iron oxide can oxidize to iron oxide (Fe_2_O_3_) or other forms of iron. At high temperatures, SiO_2_ can undergo various chemical reactions, such as the formation of various silicates. Silicates are compounds that contain silicon and oxygen and which can have different properties. A high concentration of some oxides, especially calcium oxide, can also lead to the formation of salts that can cause corrosive effects on building materials, such as concrete structures, or calcium oxide, which can react with water to form limestone, which can cause the structure to expand and warp. Water can also cause some of the minerals contained in the blast-furnace slag to dissolve, which can lead to further changes in volume. Therefore, it is important to carefully analyze a given type of slag and consider its properties and behavior in use for a given application.

On the other hand, when blast-furnace slag is in a dry environment, it does not tend to change its volume and is much more stable. Therefore, it is important that the slag is properly stored and handled in such a way as to minimize its volume changes and minimize the risks of negative effects on the structure or the environment. Examples of the use of slag in road construction were presented by [20,21]. In the proposal for the incorporation of material into the geological environment under the foundation structures, numerical analysis could be used, similar to the one presented in [22,23]. Numerical analysis requires certain soil-input data depending on the constitutive model, such as soil shear parameters and deformation parameters [24,25].

After incorporating the slag material into the subsoil, it is advisable to verify the properties of the materials, as stated by [26]. The procedure used in the evaluation of the research topic, where the concrete slab and the subsoil under the slab were tested with dynamic effects, can be applied to the layer treated with slag [27]. It would also be appropriate to use slag as a backfill material in a mechanically stabilized rock structure, as analyzed in the study by [28,29]. For a comprehensive analysis, it is necessary to know the volume of the slag material located on the site. One of the appropriate measurement methods, which was used in the creation of a digital model, was also used in the research by [30].

## 2. Materials and Methods

### 2.1. Mineralogical Composition of the Slag

The sample was subjected to X-ray powder diffractometry and RGT fluorescence spectroscopy at the Slovak Academy of Science, Institute of Geotechnics. Sample preparation included comminution and sorting on dry laboratory sieves. Shredding was carried out in three stages, of which there were two stages of crushing and one stage of grinding. After the 2nd stage of comminution, the sample was quartered, which separated the necessary amount for analysis while preserving the so-called representative amount of the sample. Subsequently, the sample was ground to an analytical fineness, i.e., a grain size below 100 μm. Control of the required grain size was carried out by sorting on a sieve with the appropriate size, while the excess product was again subjected to grinding and sorting until the entire quantity of the quartered sample did not fall through the mesh of the sieve into the collection container. Using this procedure, a tablet was prepared for the analytical sample for analyses. A tablet for XRF analysis was prepared from a finely ground sample below 100 microns [31,32,33].

The results of the elemental XRF analysis are shown in Table 1. Some elements are included in the so-called silicate analysis, which are also provided in the form of oxides. Thus, the results show that the given material mainly contains iron, silicon, and calcium, as well as aluminum, manganese, magnesium, and also the so-called alkali, i.e., sodium and potassium. Furthermore, the content of zinc, lead, and sulfur is remarkable; according to the composition, it can be assumed that the original ore was composed of chalcopyrite, sphalerite, galena, and barite, while the object of interest was probably the extraction of copper.

**Figure 2:**
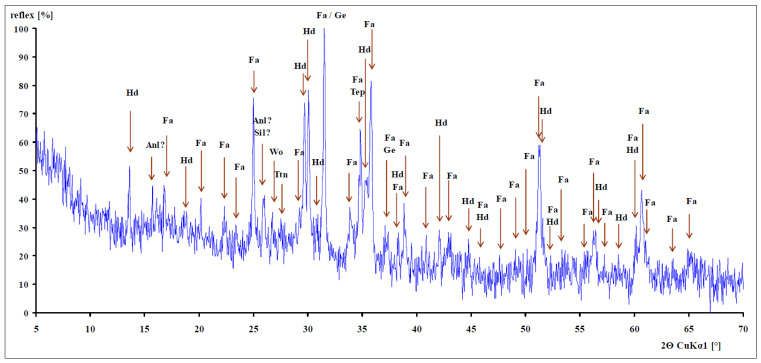
Diffractogram of a slag sample (Fa—fayalite, Hd—hedenbergite, Ge—gehlenite, Tep—tephroite, Wo—wollastonite, Sil—sillimanite, Ttn—titanite, Anl—analcin) [34].

The results of the X-ray powder diffractometry of the slag are shown in Figure 2. During the diffraction analysis, the phases often overlapped, so sometimes it was impossible to determine an unequivocal result with 100% probability (phases Anl? and Sil? in Figure 2). The main crystalline phases are fayalite Fe_2_SiO_4_, which, together with forsterite Mg_2_SiO_4_, forms the isomorphic series of olivine; olivine is the name of a mineral group of rhombic silicate minerals. Additional phases included hedenbergite CaFe_2_ + Si_2_O_6_, from the group of monoclinic pyroxenes, which forms an isomorphic series with diopside CaMgSi_2_O_6_. Further phases are also present, such as: gehlenite Ca_2_Al_2_SiO_7_, an Al-rich sorosilicate melilite group, or the isomorphic series akermanite–gehlenite Ca_2_MgSi_2_O_7_–Ca_2_Al_2_SiO_7_; tephroite Mn_2_SiO_4_ (tephroite is the manganese terminal member of the olivine group of nonsilicate minerals); wollastonite CaSiO_3_ (wollastonite is a calcium silicate mineral (CaSiO_3_) which may contain small amounts of iron, magnesium, and manganese, which replace calcium); sillimanite Al_2_SiO_5_ (sillimanite is one of the three aluminosilicate polymorphs, the other two being andalusite and kyanite); titanite CaTiSiO_5_ (a nonsilicate mineral of calcium and titanium); Anl—analcime NaAlSi_2_O_6_·H_2_O, belonging to the group of zeolites [34].

### 2.2. Size Distribution of the Slag

**Figure 3:**
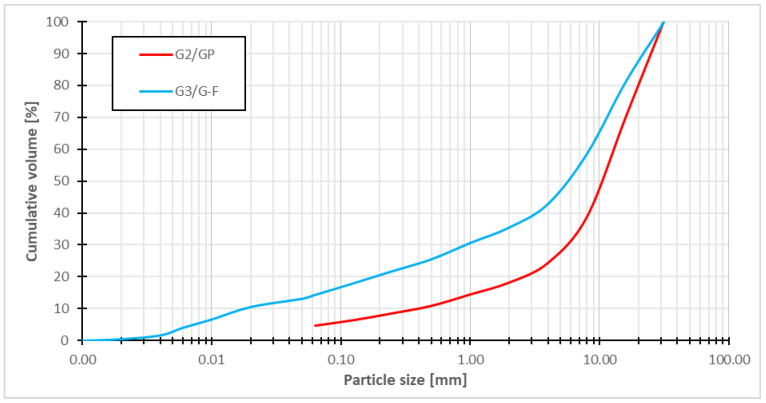
Grading curve of slag under 32 mm.

The tested blast-furnace slag from the Kremnica region was obtained as a residue from mining activity, which was described in [35]. During exploration work in Kremnica, debris was identified in the subsoil. After visual inspection, the slag was identified to be represented by two fractions, one of which had a visibly finer fraction. To ensure a representative amount, two slag samples with a minimum weight of 60 kg per sample were taken at the site. As part of the geotechnical assessment, the slag samples were sieved with a 32 mm sieve, and the slag processed in this way was subsequently used in laboratory tests. This was due to the condition of the laboratory instruments with the maximum grain tested. Then, the slag samples were sieved into the appropriate fractions and two slag grain-size curves were subsequently compiled (Figure 3).

For the red line, the grading curve had a 64.5% gravel fraction, a 21.1% sand fraction, and a 14.4% fines content. The coefficient of uniformity *C_U_* was equal to 402.6 and the coefficient of the curvature *C_C_* was equal to 4.78. For the green line, the grading curve had an 81.9% gravel fraction, a 13.5% sand fraction, and a 4.6% fines content. The coefficient of uniformity *C_U_* was equal to 33.0 and the coefficient of the curvature *C_C_* was equal to 5.55. According to STN EN ISO 14689, from the granulometric point of view, it is gravel with an admixture of fine-grained soil, *G*3*/G-F*—red line, and poorly graded gravel, *G*2*/GP*—green line.

### 2.3. Determining the Disintegrability of the Slag Aggregate by Placing It in Water

A sample of a fraction of 16/22 mm was used for the test disintegrability. Individual grains were freed from loose particles with fingers and a brush. They were then thoroughly washed with water. It was spread in a layer with a height of 1 grain on a sieve with an opening size of 16 mm, and then dried at a temperature of 105 °C to a constant weight and weighed with an accuracy of ±1 g (m_1_ = 1858.5 g). Subsequently, they were placed in a container with distilled water at a temperature of 20 ± 2 °C. After 14 days, the slag sample was removed from the water and thoroughly washed. Then, it was spread in a layer with the height of 1 grain on a sieve with an opening size of 16 mm, the sample was dried at a temperature of 105 °C to a constant weight, and then weighed with an accuracy of ±1 g (m_2_ = 1774.0 g).

From the difference in the weight of the sample before the test and after the test, a loss was determined, which was expressed in the weight of the percent with respect to the original weight of the sample. The result of the weight loss of blast-furnace slag after 14 days in water was 4.55%.

If the weight loss of the sample is greater than 5%, the slag aggregate is prone to disintegration and unsuitable for further processing. If the weight loss of the sample is less than or equal to 5%, the slag aggregate is considered to be sufficiently stable in water.

In this experiment, we focused on determining the disintegrability of the slag aggregate by placing it in water. Using this method, we find out how resistant slag aggregate is to water and whether it is suitable for specific applications where it is exposed to water.

### 2.4. Laboratory Determination of the California Bearing Ratio of Soils (CBR)

**Figure 4:**
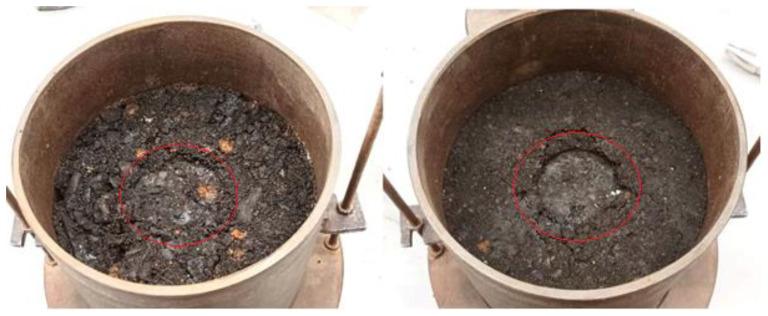
Penetration cylinder impressions inside the red circle—*CBR* test: *G*3*/G-F* is on the left and *G*2*/GP* is on the right.

Laboratory *CBR* tests were performed using the Proctor compaction method and using STN 72 1016—Laboratory determination of the California Bearing Ratio of Soil. The slag samples were sieved through a 31.5 mm sieve. A steel attachment was placed on the bottom of the container (similar to the modified Proctor) and the soil was compacted in three layers with 56 blows on each layer with a 2.5 kg hammer. Subsequently, a weight with a circular hole was placed on the surface of the sample, which simulates the weight of the structural layers of the communication. Next, a penetration roller was placed on the surface of the sample and loaded so that the total force did not exceed 50 N. This state of stress is considered initial. The penetration cylinder is pushed in with a press at a constant speed (1.00 ± 0.05) mm·min^−1^ and the force is recorded at individual steps of penetration (Table 2) from 0.5 mm to 10 mm [36,37]. The pictures (Figure 4) show the impressions of the penetration cylinder after the test.

The *CBR* is then calculated by Equation (1).

(1) $$CBR=\frac{The\;penetration\;force\;at\;2.5\;or\;5.0\;\mathrm{mm}}{Standard\;force}\cdot 100\%$$

**Figure 5:**
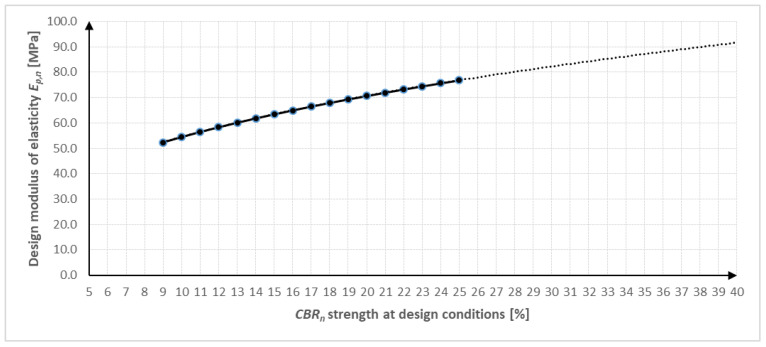
Dependence of the design modulus of elasticity *E_p,n_* on the *CBR* strength according to [38].

For the design modulus of elasticity *E_p,n_*, we take the *CBR* values at the 2.5 mm compression. From the graph (Figure 5), we can read the value of the design modulus of elasticity *E_p,n_*. After extrapolation of the field *E_p,n_* from the existing data, we obtain Equation (2). Table 3 shows the calculated *CBR* values and the *E_p,n_* values.

(2) $$E_{p,n}=22.948\cdot CBR^{0.3754}$$

The achieved values of the design modulus of elasticity *E_p,n_* = 84.1–88.1 MPa (Table 3) comply (Table 4) with class I.

### 2.5. Laboratory Determination of the Bulk Density of the Slag

Tests were performed using STN 72 1018—Laboratory determination of relative density of cohesionless soils. The 0–32 mm fraction and a container with a volume of at least 3.18 l were used for the test for the minimum and maximum bulk densities. The container was filled to about 2 cm above the top edge and the surface was leveled with a steel ruler at the top edge of the container without any compaction or shaking. The container was weighed to the nearest 0.5% of the dross. The minimum density of the soil *ρ_d,min_* was determined from two parallel determinations from the same sample, while their difference must not be greater than ±50 kg/m^3^. The lower value of the two measurements was considered to be the minimum ease. As part of the method of determining the maximum flexibility, the identical sample was continued. A guide cylinder was attached to the sampling container, a base plate was placed on the surface of the soil, and weight was lowered into the guide cylinder. The container thus prepared was firmly attached to the vibrating table and subjected to vibrations for 8 min. After the vibration stopped, the weight and the guide cylinder were removed. Subsequently, the drop of the base plate Δh was measured with a caliper. The volume of the soil in the measuring container V’ was calculated. The maximum density of the soil was determined from two parallel determinations from the same sample, while their difference must not be greater than ±50 kg/m^3^. The higher value of the two measurements was considered the maximum ease.

The difference in the tolerance of the 2 identical samples in parallel is in Δ*ρ_d,min_* = 12.3 and Δ*ρ_d,max_* = 26.4, which means that the tests are valid (Table 5). The minimum bulk density is *ρ_d,min_* = 1449.5 kg/m^3^ and the maximum bulk density is *ρ_d,max_* = 1741.7 kg/m^3^.

These tests make it possible to assess how well the material is compacted and what its bulk density is compared to the maximum and minimum values. The minimum and maximum bulk density tests allow us to assess the quality of the compaction and the compactness of the material, which is important in the design and construction of various building elements and constructions. Correctly compacted material increases its strength, durability, and resistance to deformation and barreling. These tests help ensure that the material achieves the desired properties and meets the requirements for a particular application.

### 2.6. Oedometric Test of the Slag Aggregate in a Large-Scale Shear Apparatus

This test was not done according to technical standards with prescribed dimensions and equipment, but only as a research task on a large SHEARMATIC 300 shear box apparatus with box dimensions of 30 × 30 cm and an initial sample height of 14 cm. The bulk density was chosen to be the same for both samples at 1600 kg/m^3^, which corresponds to approximately 20 kg.

After the vertical stress was applied by the hydraulic press, this vertical stress was kept constant for 2 h in the loading phase. And in the unloading phase, the vertical stress was kept constant for 1 h. The loading steps were set as follows: 0–50–100–200–400–50 kPa.

**Figure 6:**
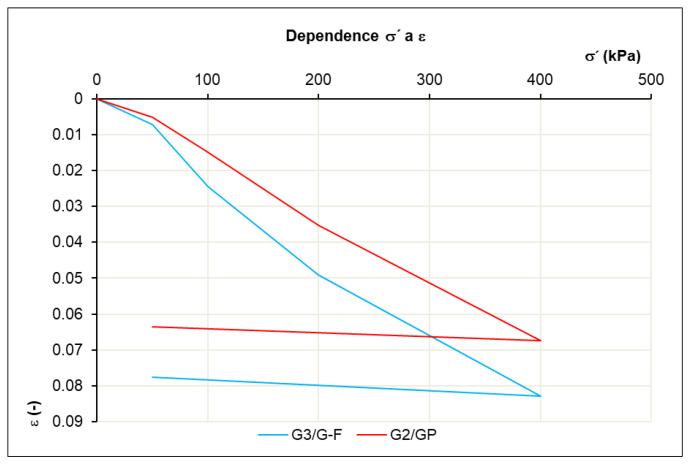
Determination of oedometric modulus *E_oed_*.

We can consider the secant of the nearby stresses *σ*_1_ and *σ*_2_ as linear, and the compressibility of the soil can be determined by the ratio Δ*σ*/Δ*ε*. The oedometric modulus of deformation *E_oed_* (3) is a secant modulus, valid for a certain stress interval (Table 6) on the deformation curve (Figure 6), where we plot the stress on the x-axis and the deformation on the y-axis.

(3) $$E_{oed}=\frac{\Delta \sigma }{\Delta \varepsilon }=\frac{\sigma_{2}-\sigma_{1}}{\varepsilon_{2}-\varepsilon_{1}}$$

The results of the soil oedometric modules are important in the design of geotechnical structures, such as building foundations, embankments, road subgrades, etc. They help engineers to understand the behavior of the soil under load and make it possible to predict deformations and stability in specific geotechnical conditions.

### 2.7. Laboratory Determination of the Shear Strength of the Slag Aggregate in a Box Apparatus

**Figure 7:**
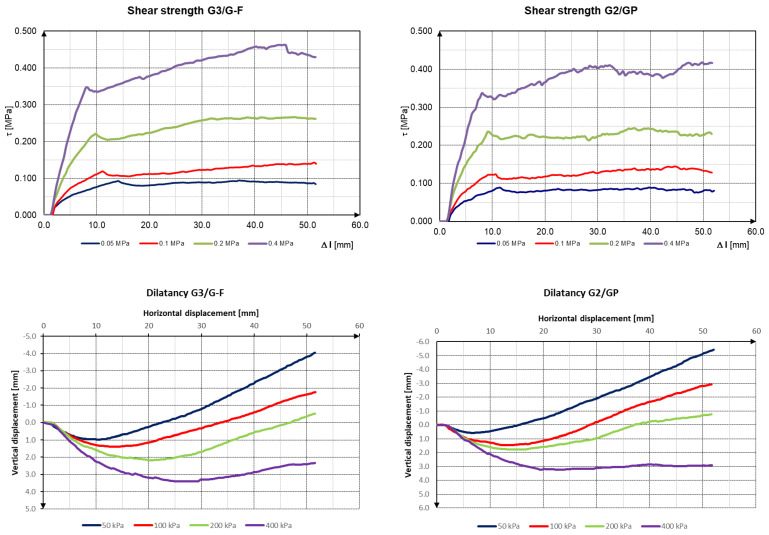

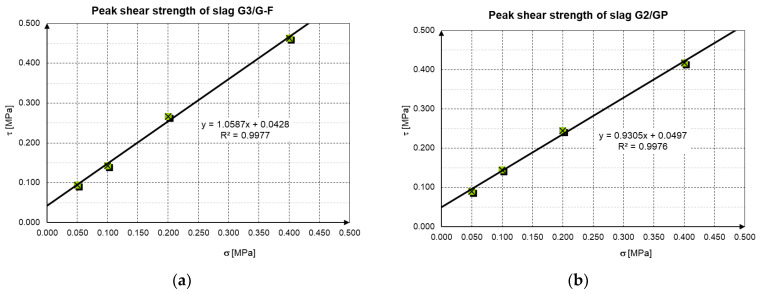
Evaluation of shear box tests of blast-furnace slag. (**a**) *G*3*/G-F*; (**b**) *G*2*/GP*.

The slag sample was tested in a large SHEARMATIC 300 shearing machine with box dimensions of 30 × 30 cm and a sample height of 14 cm. The sliding speed of the sample was set at 0.025 mm/min. Tests were performed using STN 72 1030—Laboratory direct shear box drained test of soils. For a better comparison, the same volume weight was chosen for both samples, namely 1600 kg/m^3^, which corresponds to approximately 20 kg. Two mixtures of *G*3*/G-F* and *G*2*/GP* slag were tested. The tension intervals were set at 50–100–200–400 kPa. At these vertical stresses *σ*, the maximum shear stresses τ were reached, which are plotted in the figure (Figure 7) [39,40].

We can see (Figure 7) the shear box displacement/shear stress plot is more scattered in the case of sample *G*2*/GP* than that of sample *G*3*/G-F*. This is caused by the structure of the material, where the individual cavities in the *G*3*/G-F* sample are more filled with finer material. In Figure 7, we also see dilatancy curves; the slag samples increased their volume with increasing shear. For the practical purposes of designing building structures, the dilatancy angle *ψ* is given by Equation (4) [41,42,43]. Excellent determination coefficients of *R*^2^ = 0.997 were achieved for both slag samples. For sample *G*3*/G-F*, the angle of internal friction was *φ* = 46.6° and the cohesion was *c* = 42.8 kPa. For sample *G*2*/GP*, the angle of internal friction was *φ* = 42.9° and the cohesion was *c* = 49.7 kPa.

(4) ψ=φ−30°

## 3. Results and Discussion

Studying the subject sample determined the main crystalline phases of fayalite and hedenbergite, with a high content of iron, silicon, and calcium. According to the geotechnical evaluation, the slag samples were classified as *G*3*/G-F* and *G*2*/GP*. Based on the disintegration test in water, the result of the weight loss of the blast-furnace slag after 14 days in water was 4.55%, so the slag sample did not reach the limit of 5% and would not be suitable for further processing.

The *CBR* test achieved favorable results of 36.0% and 31.8%. These *CBR* values indicate that the blast-furnace slag has a good bearing capacity and strength under load. This is advantageous because it means the material is able to withstand loads used in a variety of geotechnical applications, such as road foundations, parking lots, embankments, and so on. These results are in agreement with the achieved first-traffic-load class, which is defined through the design modulus of elasticity *E_p,n_* > 60 MPa. The values of the design modulus of elasticity *E_p,n_* reached 84 and 88 MPa, respectively.

The relative-lightness test determined that the minimum volume weight is *ρ_d,min_* = 1449.5 kg/m^3^ and the maximum volume weight is *ρ_d,max_* = 1741.7 kg/m^3^. These tests make it possible to assess how well the material is compacted and what its bulk density is compared to the maximum and minimum values. The minimum and maximum volumetric weight tests allow us to assess the quality of the compaction and compactness of the material, which is important in the design and construction of various building elements and constructions. Properly compacted material increases its strength, durability, and resistance to deformation and damage. These tests help ensure that the material achieves the desired properties and meets the requirements set for the specific application.

The oedometric test achieved module ranges of *E_oed_* = 4.0–6.5 MPa at a stress interval of 100–400 kPa. Lower oedometric modules were achieved by the unique structure of the material. With natural materials, we achieve orders of magnitude higher oedometric modules. The oedometric test, also known as the compressibility test, is an important laboratory test in geotechnics. It shows the behavior of soil under load.

The resulting values of the angle of internal friction and cohesion as the basic shear parameters of soils were achieved by the box test. Without these shear parameters, one could not proceed with many geotechnical problems. Analyzing the test output, the resulting values of the angle of internal friction, angle of dilatancy, and values of cohesion can be obtained. For sample slag *G*3*/G-F*, the angle of internal friction was *φ* = 46.6 ° and the cohesion was *c* = 42.8 kPa. For the sample slag *G*2*/GP*, the angle of internal friction was *φ* = 42.9° and the cohesion was *c* = 49.7 kPa.

## 4. Conclusions

The study was focused on determining the basic geotechnical parameters for blast-furnace slag as a product of mining activity in the Kremnica region. The main task of this case study was the proper evaluation of the soil subgrade composed from furnace slag from the point of view of its deformability, stability through the time, and environmental impact; this means that the soil subgrade with artificial layers of furnace slag can be stable through the lifetime of earth structures and not contaminate the soil environment after construction arrangements, and possibly change the hydraulic regime of the groundwater. In the past, inappropriate evaluation of furnace slag as a material substitute for crushed aggregate layers caused serious deformations on the D1 motorway near Ostrava, Czech Republic, shopping mall floor deformations, and many other negative examples can be stated. Furnace slag in these cases was in contact with water swells and increased in volume. After absorbing water into the structure, the disintegration process of solid particles is rapid and, thanks to a loss of strength, produces deformations that can be in decimeters [44]. The Department of Geotechnics of the University of Zilina has provided many years of research about foamed-concrete-composite structures [44]. This can be a good alternative for remediation in cases where massive deformation due to structural collapse of furnace-sludge fillings occurred.

The advantages are in the easy filling of empty spaces, the short time-period of strengthening, and the variability in the parameter settings according to the task of use [45]. Nowadays, the green strategy of using more recycled and waste materials in the construction sector is necessary, but with nonproper evaluation of these materials, economic and environmental losses can be high. To achieve this goal, we performed an extensive series of tests, including mineralogical composition, size distribution, disintegration of the aggregates placed in water, the minimum and maximum bulk densities, the California bearing ratio (*CBR*), the oedometric test, and the shear test.

Our results show that the slag reached a disintegration value lower than 5%, which makes it a suitable material for use in geotechnical construction. From the point of view of the *CBR* and *E_p,n_*, we have classified the slag so that it can withstand the load of the I class of transport. Through the tests, we identified the min and max. slag density, while it was proved that the maximum density is low due to the high porosity of the slag.

The shear box displacement/shear stress plot is more scattered in the case of sample *G*2*/GP* than that of sample *G*3*/G-F*. This is caused by the structure of the material, where the individual cavities in the *G*3*/G-F* sample are more filled with a finer material. From the curves, we can obtain the resulting values of the angle of the internal friction, the angle of dilatancy, and the values of cohesion. For the sample slag *G*3*/G-F*, the angle of internal friction was *φ* = 46.6° and the cohesion was *c* = 42.8 kPa. For the sample slag *G*2*/GP*, the angle of internal friction was *φ* = 42.9° and the cohesion was *c* = 49.7 kPa. Regarding the deformation parameters, we set the *E_oed_* in the stress interval of 100–400 kPa to values of *E_oed_* = 4–6.5 MPa. The unique structure of the slag material provided different measures of basic geotechnical parameters compared to the natural material of similar grain size. For expanding our knowledge about slag, we recommend statistical processing, which can be the subject of further scientific work.

The presented study makes it possible to use this material in geotechnical construction. Proper management of blast-furnace slag from metal processing is essential to minimize the environmental impact and ensure sustainability in the mining industry. From an environmental, material, and, consequently, economic point of view, their recovery on an industrial scale is more advantageous.

## Figures and Tables

**Table 1:** XRF analysis—basic elements (gray), metals (red), and trace elements (yellow).

Element	Content (%)	Element	Content (%)	Element	Content (%)	Element	Content (ppt)	Element	Content (ppt)	Element	Content (ppt)
Fe_2_O_3_	37.74	MnO	1.26	Zn	2.88	Sb	238	Sn	111	Hg	39
SiO_2_	29.61	K_2_O	1.23	Pb	2.64	Zr	179	Rb	90	Mo	28
CaO	8.12	MgO	0.74	S	0.89	Sr	177	Cd	84	Se	23
Al_2_O_3_	2.90	TiO_2_	0.32	Ba	0.21	Te	154	V	60	Br	18
Na_2_O	1.29	P_2_O_5_	0.19	Cu	0.13	Ag	151	Ni	46	As	0.2

**Table 2:** Measured values of the *CBR* test.

Measured *CBR* Test Values for Sample no. 1 (*G*3*/G-F*)											
Mandrel penetration depth (mm)	0	0.5	1	1.5	2	**2.5**	3	4	**5**	7.5	10
Force (kN)	0	1.6	2.5	3.3	4.05	**4.75**	5.2	6.5	**7.7**	9.85	11.9
**Measured *CBR* test values for sample no. 2 (*G*2*/GP*)**											
Mandrel penetration depth (mm)	0	0.5	1	1.5	2	**2.5**	3	4	**5**	7.5	10
Force (kN)	0	1.2	2.2	2.95	3.6	**4.2**	4.5	5.55	**6.2**	7.1	8.4

**Table 3:** Achieved *CBR* and *E_p,n_* values.

Penetration (mm)	Standard (kN)	Sample no.1 (kN)	Sample no.2 (kN)	*CBR* no.1 (%)	*CBR* no.2 (%)	*E_p,n_* no.1 (MPa)	*E_p,n_* no.2 (MPa)
2.5	13.2	4.75	4.2	**36.0**	**31.8**	88.1	84.1
5.0	20	7.7	6.2	38.5	31.0		

**Table 4:** Characteristics of the standard conditions in the subsoil for the Slovak Republic [38].

Traffic Load Classes	*E_p,n_* (MPa)
(The class I) *E_p,n_* for heavy traffic loads	≥60
(The class II a III) *E_p,n_* for medium traffic loads	≥40
(The class IV až VI) *E_p,n_* for small traffic loads	≥30

**Table 5:** Minimum and maximum bulk densities of the slag.

Test	m_1_ (kg)	V (m^3^)	Δh (mm)	Δhp (mm)	V’ (m^3^)	*ρ_d,min_* (kg/m^3^)	*ρ_d,max_* (kg/m^3^)
1.	4.65	0.00318	29.5	26.075	0.00271	1461.8	1715.2
			24.2				
			23.0				
			27.6				
2.	4.61	0.00318	29.6	29.600	0.00265	1449.5	1741.7
			28.5				
			30.1				
			30.2				

**Table 6:** Calculated values *E_oed_* for various stress intervals.

Stress Interval (kPa)		0–50	50–100	100–200	200–400	400–50
*G*3*/G-F*	*E_oed_* (MPa)	7.01	2.89	4.05	5.93	68.27
*G*2*/GP*		9.51	5.21	4.88	6.24	92.65

## References

[1] Bastidas-Martínez J.G., Reyes-Lizcano F.A., Rondón-Quintana H.A. (2022). Use of recycled concrete aggregates in asphalt mixtures for pavements: A review. J. Traffic Transp. Eng. (Engl. Ed.).

[2] Buczyński P., Šrámek J., Mazurek G. (2023). The Influence of Recycled Materials on Cold Mix with Foamed Bitumen Properties. Materials.

[3] Sas W., Dzięcioł J., Radzevičius A., Radziemska M., Dapkienė M., Šadzevičius R., Skominas R., Głuchowski A. (2021). Geotechnical and Environmental Assessment of Blast Furnace Slag for Engineering Applications. Materials.

[4] Cajka R., Martinec P. (2011). Structural failures of buildings caused by volume changesof steel slag. Trans. VSB-Tech. Univ. Ostrav..

[5] Coomarasamy A., Walzak T.L. (1995). Effects of moisture on surface chemistry of steel slags and steel slag-asphalt paving mixes. Transp. Res. Rec..

[6] Shi W.Y., Shao H.B., Li H., Shao M.A., Du S. (2009). Progress in the remediation of hazardous heavy metal-polluted soils by natural zeolite. J. Hazard. Mater..

[7] Angelovičová L., Fazekašová D. (2014). Contamination of the soil and water environment by heavy metals in the former mining area of Rudňany (Slovakia). Soil Water Res..

[8] Fazekašová D., Fazekaš J. (2020). Soil Quality and Heavy Metal Pollution Assessment of Iron Ore Mines in Nizna Slana (Slovakia). Sustainability.

[9] Angelovičová L., Bobuľská L., Fazekašová D. (2015). Toxicity of heavy metals to soil biological and chemical properties in conditions of environmentally polluted area middle Spiš (Slovakia). Carpathian J. Earth Environ. Sci..

[10] Štofejová L., Fazekaš J., Fazekašová D. (2021). Analysis of Heavy Metal Content in Soil and Plants in the Dumping Ground of Magnesite Mining Factory Jelšava-Lubeník (Slovakia). Sustainability.

[11] Demková L., Árvay J., Bobuľská L., Tomáš J., Stanovič R., Lošák T., Jobbágy J. (2017). Accumulation and environmental risk assessment of heavy metals in soil and plants of four different ecosystems in a former polymetallic ores mining and smelting area (Slovakia). J. Environ. Sci. Health.

[12] Mark O., Ede A., Arum C., Oyebisi S. (2021). Effects of Induction-Furnace Slag on Strength Properties of Self-Compacting Concrete. Civ. Environ. Eng..

[13] Ali Z.H., Khalid N.N. (2023). Applicability of Induction Furnace Steel Slag in RC Columns Subjected to Axial and Uniaxial Loading. Civ. Environ. Eng..

[14] Juang C.-U., Kuo W.-T. (2023). Properties and Mechanical Strength Analysis of Concrete Using Fly Ash, Ground Granulated Blast Furnace Slag and Various Superplasticizers. Buildings.

[15] Nicula L.M., Manea D.L., Simedru D., Cadar O., Becze A., Dragomir M.L. (2023). The Influence of Blast Furnace Slag on Cement Concrete Road by Microstructure Characterization and Assessment of Physical-Mechanical Resistances at 150/480 Days. Materials.

[16] Özbay E., Erdemir M., Durmuş H.İ. (2016). Utilization and efficiency of ground granulated blast furnace slag on concrete properties–A review. Constr. Build. Mater..

[17] Baricová D., Pribulová A., Demeter P., Buľko B., Rosová A. (2012). Utilizing of the metallurgical slag for production of cementless concrete mixtures. Metalurgija.

[18] Hredzák S., Matik M., Šestinová O., Zubrik A., Hančuľák J., Dolinská S., Znamenáčková I. (2021). Identification of blast furnace slag in applications of road construction. (Identifikácia vysokopecnej trosky pri aplikáciách v cestnom staviteľstve). SZVK.

[19] Michalíková F., Jacko V., Sisol M., Kozáková Ľ. (2005). Thermal power sludge—Properties, treatment, utilization. (Teplárenská troska–vlastnosti, úprava a použitie). Acta Montan. Slovaca.

[20] Vukićević M., Marjanović M., Pujević V., Jocković S. (2019). The Alternatives to Traditional Materials for Subsoil Stabilization and Embankments. Materials.

[21] Nawagamuwa U., Madushanka H., Katubedda M. Ground Improvement with Waste Copper Slag. Proceedings of the 4th International Symposium on Advances in Civil and Environmental Engineering.

[22] Decký M., Drusa M., Papán D., Šrámek J. (2022). The Relationship between Dynamic and Static Deformation Modulus of Unbound Pavement Materials Used for Their Quality Control Methodology. Materials.

[23] Vlcek J., Valaskova V. (2019). Investigation of the soilgeosynthetic interaction using direct shear testing and FEM method. IOP Conf. Ser. Mater. Sci. Eng..

[24] Stacho J., Sulovska M. (2022). Shear Strength Properties of Coarse-Grained Soils Determined Using Large-Size Direct Shear Test. Civ. Environ. Eng..

[25] Stacho J., Sulovska M., Slavik I. (2023). Analysis of the Shear Strength of a Soil-Geosynthetic Interface. Civ. Environ. Eng..

[26] Drusa M., Vlček J., Orininová L. (2016). The Role of Geotechnical Monitoring at Design of Foundation Structures and their Verification—Part 1. Civ. Environ. Eng..

[27] Valašková V., Vlček J. (2018). Stress Response Analysis of Concrete Pavement Under Tire of Heavy Vehicle. Civ. Environ. Eng..

[28] Drusa M., Vlček J., Holičková M., Kais L. (2016). Analytical and Numerical Evaluation of Limit States of MSE Wall Structure. Civ. Environ. Eng..

[29] Valašková V., Vlček J., Papán D. (2020). Determination of the Small-Scale Physical Model Parameters of Pavement Structure. Sustainability.

[30] Muzik J., Seidlova A., Kudelcikova M., Kongar-Syuryun C., Mihalik J. (2021). Flood hazard calculation by using a digital terrain model. IOP Conf. Ser. Earth Environ. Sci..

[31] Zubrik A., Matik M., Lovás M., Danková Z., Kaňuchová M., Hredzák S., Briančin J., Šepelák V. (2019). Mechanochemically Synthesised Coal-Based Magnetic Carbon Composites for Removing As(V) and Cd(II) from Aqueous Solutions. Nanomaterials.

[32] Längauer D., Čablík V., Hredzák S., Zubrik A., Matik M., Danková Z. (2021). Preparation of Synthetic Zeolites from Coal Fly Ash by Hydrothermal Synthesis. Materials.

[33] Znamenáčková I., Dolinska S., Hredzak S., Čablík V., Lovas M., Gešperová D. (2020). Study of Extraction of Rare Earth Elements from Hard Coal Fly Ash. Inż. Miner..

[34] Hredzák S., Matik M., Findoráková L., Šestinová O., Zubrik A., Dolinská S., Znamenáčková I. Analysis of the historical slag from kremnica. Proceedings of the GEOCHEMIA 2023, Bratislava—State Geological Institute of Dionýz Štúr.

[35] Števko M., Sejkora J., Dolníček Z., Škácha P. (2018). Selenium-Rich Ag–Au Mineralization at the Kremnica Au–Ag Epithermal Deposit, Slovak Republic. Minerals.

[36] Trong D.K., Pham B.T., Jalal F.E., Iqbal M., Roussis P.C., Mamou A., Ferentinou M., Vu D.Q., Duc Dam N., Tran Q.A. (2021). On Random Subspace Optimization-Based Hybrid Computing Models Predicting the California Bearing Ratio of Soils. Materials.

[37] López A., Mayacela M., Chérrez D., Aldas E., Contreras L.F. (2023). Comparison of Physical and Mechanical Properties of Stone Aggregates and Their Use in the Structure of a Flexible Pavement, from Mines in Ecuador. Buildings.

[38] (2009). Design of Non-Rigid and Semi-Rigid Roadway.

[39] Nhema C.C., Ke H., Ma P., Chen Y., Zhao S. (2021). The Influence of Discrete Fibers on Mechanical Responses of Reinforced Sand in Direct Shear Tests. Appl. Sci..

[40] Daghistani F., Baghbani A., Abuel Naga H., Faradonbeh R.S. (2023). Internal Friction Angle of Cohesionless Binary Mixture Sand–Granular Rubber Using Experimental Study and Machine Learning. Geosciences.

[41] Akram I., Azam S. (2023). Effect of Sample Preparation on Saturated and Unsaturated Shear Strength of Cohesionless Soils. Geotechnics.

[42] Chen J., Zhang Y., Yang Y., Yang B., Huang B., Ji X. (2023). Influence of Coarse Grain Content on the Mechanical Properties of Red Sandstone Soil. Sustainability.

[43] Benjelloun M., Bouferra R., Ibouh H., Jamin F., Benessalah I., Arab A. (2021). Mechanical Behavior of Sand Mixed with Rubber Aggregates. Appl. Sci..

[44] Vlcek J., Drusa M., Scharfel W., Sedlar B. Experimental Investigation of Properties of Foam Concrete for Industrial Floors in Testing Field. Proceedings of the 3rd World Multidisciplinary Earth Sciences Symposium.

[45] Hajek M., Decky M., Drusa M., Orininova L., Scherfel W. Elasticity Modulus and Flexural Strength Assessment of Foam Concrete Layer of Poroflow. Proceedings of the 3rd World Multidisciplinary Earth Sciences Symposium.

